# Role of long non-coding RNA in chemoradiotherapy resistance of nasopharyngeal carcinoma

**DOI:** 10.3389/fonc.2024.1346413

**Published:** 2024-02-29

**Authors:** Yang Yang, QuPing Yuan, Weijian Tang, Ya Ma, JingYan Duan, GuoNing Yang, Yuan Fang

**Affiliations:** ^1^ Otorhinolaryngology Head and Neck Surgery, Baoshan People’s Hospital, Baoshan, Yunnan, China; ^2^ Puer People’s Hospital, Department of Critical Medicine, PuEr, Yunnan, China; ^3^ Queen Mary School of Nanchang University, Nanchang University, Nanchang, China; ^4^ Department of Organ Transplantation, The First Affiliated Hospital of Kunming Medical University, Kunming, Yunnan, China

**Keywords:** nasopharyngeal carcinoma, long noncoding RNAs, drug resistance, radiation resistance, recurrence

## Abstract

Nasopharyngeal carcinoma (NPC) is a malignant tumor originating from the nasopharyngeal epithelial cells. Common treatment methods for NPC include radiotherapy, chemotherapy, and surgical intervention. Despite these approaches, the prognosis for NPC remains poor due to treatment resistance and recurrence. Hence, there is a crucial need for more comprehensive research into the mechanisms underlying treatment resistance in NPC. Long non coding RNAs (LncRNAs) are elongated RNA molecules that do not encode proteins. They paly significant roles in various biological processes within tumors, such as chemotherapy resistance, radiation resistance, and tumor recurrence. Recent studies have increasingly unveiled the mechanisms through which LncRNAs contribute to treatment resistance in NPC. Consequently, LncRNAs hold promise as potential biomarkers and therapeutic targets for diagnosing NPC. This review provides an overview of the role of LncRNAs in NPC treatment resistance and explores their potential as therapeutic targets for managing NPC.

## 1 Introduction

Nasopharyngeal carcinoma (NPC) is a malignant tumor originating in the epithelial cells of the nasopharynx (1). Although NPC has been linked to various factors, such as viral infections, environmental influences, and genetics, its precise pathogenesis remains unclear. At present, the latest research suggests that NPC is not caused by a single factor, but a unified disease of ecology and evolution, and cancer cells and tumor microenvironment (TME) constitute a complex pathological ecosystem (2). The global incidence of NPC varies significantly, with higher rates observed in high-risk regions. In areas such as South China and Hong Kong, the incidence can reach 25-50 cases per 100,000 people, while in the Western world, NPC relatively rarely occurs, with an annual incidence of less than one in 100,000 people (3, 4). In low-risk groups, NPC incidence is positively correlated with age. However, in medium and high-risk groups, the peak incidence of NPC occurs among the youth and those aged 45-54 years, with a decline in NPC cases among older individuals (3). Moreover, men are 2-3 times more likely than women to develop NPC (1). Early-stage NPC often presents nonspecific symptoms, leading to potential misdiagnosis. Many NPCs were not detected until the intermediate and late stages. Because of its hidden anatomical location and high sensitivity to radiotherapy, surgical treatment for NPC is not the primary choice; instead, radiotherapy remains the mainstay. Although most patients with NPC undergo chemoradiotherapy, resistance to this treatment is common, leading to very high recurrence and metastasis rates and low overall survival (OS). Approximately 10% of patients with NPC experience recurrence, while 10%-20% develop distant metastasis (5). Globally, the OS rate for patients with NPC is less than 80%, with a 5-year OS rate of 51.5% for stage III NPC and as low as 32.4% for stage IVA NPC (6). Consequently, due to the late detection, high recurrence rates, and unsatisfactory treatment outcomes, there is an urgent need for extensive research into NPC pathogenesis to improve diagnosis and treatment.

**Figure 1:**
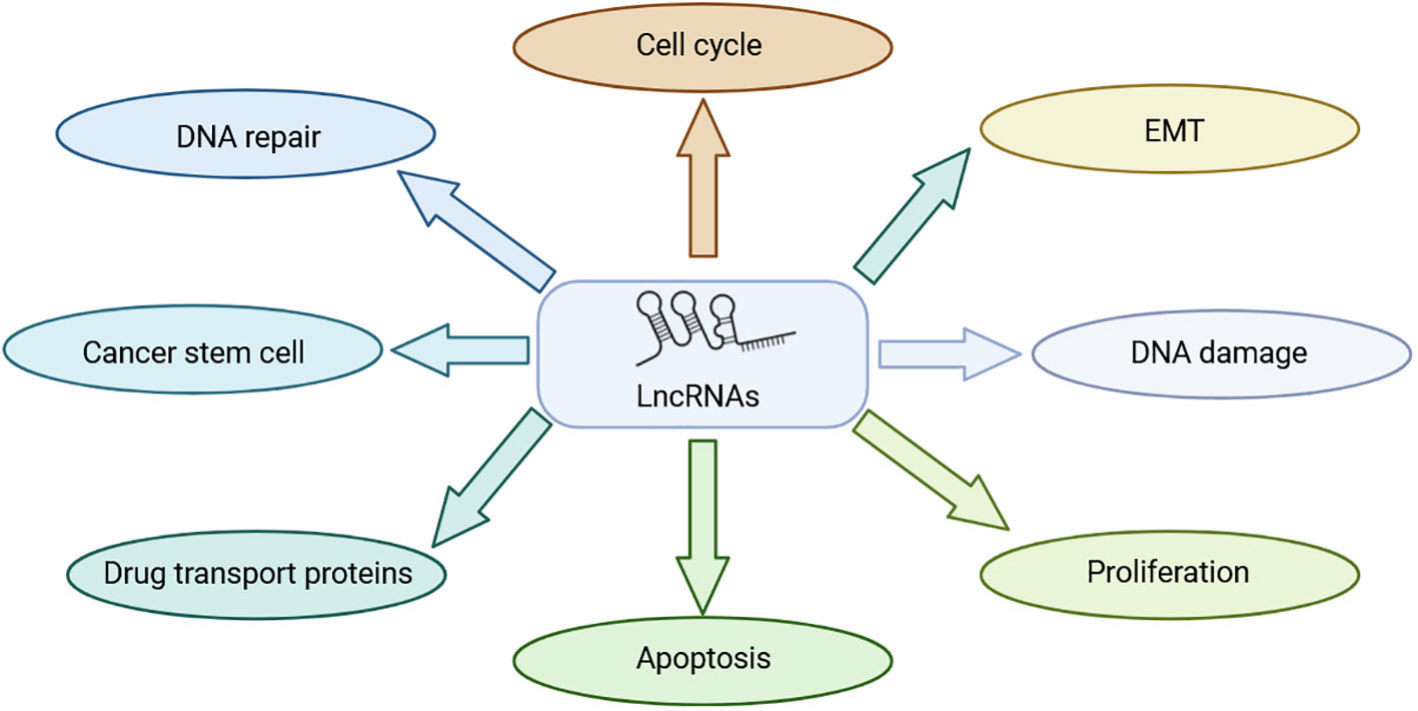
Role of LncRNA in NPC treatment resistance.

**Figure 2:**
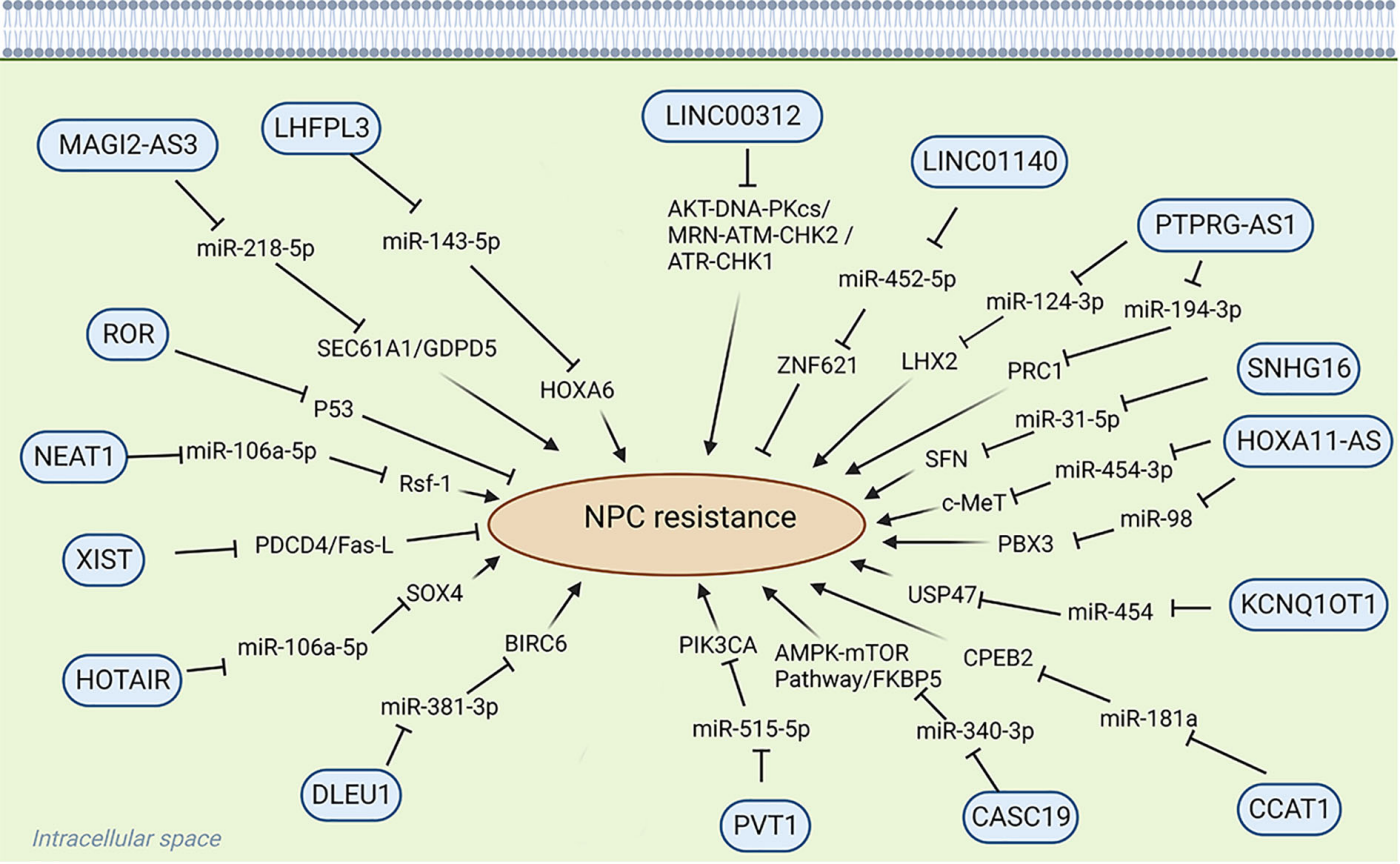
Role of LncRNA in chemoradiotherapy resistance.

Long noncoding RNAs (LncRNAs) are transcripts exceeding 200 nucleotides that cannot encode proteins (7). They play a very important role in physiological and pathological regulation (8) (**Figure 1**). Because LncRNAs lack protein-coding ability, it was believed that they die not have a role in the physiological and pathological processes of the body, thus receiving less attention from researchers (9). Historically overlooked, recent years have seen a shift in understanding their significance in various physiological and pathological processes, notably in tumor initiation and progression. LncRNAs have shown substantial involvement in chemoradiotherapy resistance in NPC. For instance, LHFPL3 is implicated in regulating NPC radiotherapy resistance by modulating the expression of the target gene HOXA6 through sponge miR-143-5p, thereby regulating NPC sensitivity to radiotherapy; this suggests that LHFPL3 may serve as a potential target in NPC treatment (10). NHG16 is highly expressed in NPC cells, and sponge miR-31-5p is believed to enhance the expression of the target gene SFN, thereby enhancing the radioresistance of NPC (11). However, the exact function of NHG16 is not fully understood. KCNQ1OT1, another LncRNA, significantly influences NPC drug resistance. KCNQ10T1 knockout promotes chemotherapy sensitivity of NPC cells through the miR-454/USP47 axis, offering a potential solution to chemotherapy resistance in patients with NPC (12). While the precise mechanism of LncRNA in mediating NPC chemoradiotherapy resistance remains unclear, current studies highlight its potential as a therapeutic target for NPC chemoradiotherapy resistance. This review introduces the role of LncRNAs in NPC chemoradiotherapy resistance and summarizes LncRNAs associated with NPC resistance (**Figure 2**) (**Table 1**).

**Table 1:** LncRNAs involved in NPC resistance.

NcRNAs	Location	Expression	Target	Resistant type	Role in drug resistance	Functions	References
LHFPL3	7q22.2-q22.3	↑	HOXA6	Radiotherapy	Promoting	proliferation, apoptosis, migration, invasion	(10)
SNHG16	17q25.1	↑	SFN	Radiotherapy	Promoting	proliferation	(11)
KCNQ1OT1	11p15.5	↑	USP47	Chemotherapy	Promoting	Proliferation, migration, invasion	(12)
DLEU1	13q14.2-q14.3	↑	BIRC6	Chemotherapy	Promoting	proliferation	(13)
HOTAIR	12q13.13	↑	miR-106a-5p/SOX4	Chemotherapy	Promoting	Apoptosis, migration, invasion	(14)
XIST	Xq13.2	↑	PDCD4, Fas-L	Chemotherapy	Promoting	Proliferation, apoptosis, migration, invasion	(15, 16)
ROR	18q21.31	↑	P53 signaling pathway	Chemotherapy	Promoting	Proliferation, apoptosis, migration	(17–19)
NEAT1	11q13.1	↑	Rsf-1/Bcl-2	Chemotherapy	Promoting	proliferation, migration, invasion	(20, 21)
MAGI2-AS3	7q21.11	↑	GDPD5,SEC61A1	Chemotherapy	Promoting	proliferation, migration	(22)
HOXA11-AS	7p15.2	↑	c-MeT/PBX3	Chemotherapy	Promoting	Proliferation, apoptosis	(23, 24)
CCAT1	8q24.21	↑	CPEB2	Chemotherapy	Promoting	proliferation, apoptosis, migration, invasion	(25)
PVT1	8q24.21	↑	PIK3CA	Radiotherapy	Promoting	Proliferation, apoptosis	(26, 27)
LINC01140	1p22.3	↓	ZNF621	Radiotherapy	Promoting	Proliferation, apoptosis	(28)
Linc00312	3p25.3	↓	AKT-DNA-PKcs, MRN-ATM-CHK2, ATR-CHK1	Radiotherapy	Promoting	Proliferation, invasion	(29, 30)
PTPRG-AS1	3p14.2	↑	MiR-124-3p/LHX2,miR-194-3p/PRC1	Radiotherapy	Promoting	Proliferation, apoptosis, migration, invasion	(31, 32)
CASC19	8q24.21	↑	AMPK-mTOR Pathway/FKBP5	Radiotherapy	Promoting	proliferation apoptosis, migration, invasion	(33, 34)

## 2 Role of IncRNA in NPC chemotherapy resistance

Platinum drugs are cytotoxic drugs used as first-line chemotherapeutic agents for many cancers. Their mode of action involves binding to DNA after hydrolyzing one or two chloride ions in the body, thereby impeding replication and transcription and, consequently, the proliferation of cancer cells (35, 36). However, prolonged usage of platinum-based drugs may lead to the development of resistance, which can compromise treatment efficacy. The mechanisms underlying drug resistance to platinum drugs in malignant tumors include several facets: First, overexpression of drug efflux transporters diminishes intracellular drug accumulation. Second, increased DNA repair diminishes sensitivity to chemotherapy. Third, intracellular drug inactivation also contributes to this resistance (37, 38).Recent studies have emphasized the significant role of LncRNA in tumor chemotherapy resistance (13). For instance, in NPC tissues, upregulated expression of LncRNA DLEU1 enhances the expression of BIRC6 through the sponge miR-381-3p, thereby promoting cisplatin resistance in NPC cells (13). Competitive endogenous RNA (ceRNA) is a novel mechanism for regulating gene expression. LncRNAs with the same miRNA response elements competitively bind to the same miRNA, thus diminishing its inhibition of target gene mRNA and modulating NPC development (39). HOTAIR, highly expressed in cisplatin-resistant cells, regulates target gene expression through ceRNA. Knockdown of HOTAIR can reduce cell viability, expression, migration and invasion of chemical resistance related proteins, increase cell apoptosis, reduces the expression of target gene SOX4 through miR-106a-5p, heightening the chemosensitivity of NPC cells to cisplatin. This suggests that HOTAIR is a potential therapeutic target in NPC therapy (14). LINC00346, located on chromosome 13q34, has shown abnormal expression in various diseases including NPC. Its aberrant expression significantly correlates with tumor proliferation, invasion, and drug resistance, establishing it as a potential biomarker for disease diagnosis and treatment (40). There is no doubt that chemotherapy sensitivity of malignant tumor cells is closely related to cell proliferation and apoptosis (41). XIST, an important mammalian LncRNA derived from the XIST gene, upregulated in NPC cisplatin cells, decreases the expression of programmed cell death 4 (PDCD4) and Fas ligand (Fas-L), thereby increasing drug resistance and reducing chemosensitivity in NPC cells (15, 16). ROR, an oncogenic LncRNA located on chromosome 18, is overexpressed in NPC. Studies have shown that the enrichment of ROR is related to the chemotherapy resistance of NPC. P53 is one of the important tumor suppressors in the body. ROR inhibits the P53 signaling pathway, which may elucidate the mechanism of NPC resistance to chemotherapy, indicating the pivotal role of ROR in NPC progression (17–19). It could serve as a potential therapeutic target to mitigate chemotherapy resistance in NPC. NEAT1 up-regulates the expression of Rsf-1 through let-7a-5p, thereby enhancing the resistance of NPC cells to cisplatin. In addition, NEAT1 can also regulate the resistance of nasopharyngeal carcinoma to cisplatin through the regulation of Ras-MAPK signaling pathway (20). Epithelial-mesenchymal transition (EMT) is a complex process that can regulate the changes of cell morphology and function during embryogenesis and tissue development, and tumor cells can obtain drug resistance through EMT (42, 43). MAGI2-AS3 can regulate the expression of target genes GDPD5 and SEC61A1 through sponge miR-2185p, thereby regulating the proliferation, migration and EMT of NPC cells, as well as the cisplatin resistance of NPC (22). HOXA11-AS is pivotal in various cancers such as lung adenocarcinoma (44), oral squamous cell carcinoma (45), gastric cancer (46), prostate cancer (47), and breast cancer (48) and is closely related to NPC. HOXA11-AS is increasingly expressed in cisplatin-resistant NPC cells and knocking down HOXA11-AS could increase the expression of miR-454-3p and inhibit the expression of target gene c-Met, thereby increasing the sensitivity of the tumor cells to cisplatin (23). Knocking out HOXA11-AS can also decrease the expression of the target gene PBX3 through sponge miR-98, further increasing the sensitivity of NPC cells to cisplatin. These findings highlight a potential target for treating drug-resistant NPC (24).

Paclitaxel, derived from the Pacific redwood, is the first approved herb-derived chemotherapeutic drug (49, 50), and is widely used in the treatment of many types of cancer. Paclitaxel binds to microtubules, disrupting microtubule protein depolymerization. This interference inhibits mitosis, ultimately leading to the death of cancer cells (51, 52). Although paclitaxel is effective in treating malignant tumors, resistance to paclitaxel remains a significant impediment to its effectiveness. The exact mechanisms underpinning paclitaxel resistance are presently unclear. However, certain studies propose its association with altered microdynamics, changes in micro-protein expression, and modifications in tumor- suppressing signaling pathways (51). H19, known as the imprinted maternally expressed transcript, represents one of the earliest identified LncRNAs. It is located in the 11p15.5 chromosomal region and is encoded by the H19 gene (53, 54). Accumulating evidence underscores the pivotal role of H19 in the progression and drug resistance of malignant tumors, rendering it a focal point of research. Studies have identified its resistance to various anticancer drugs such as paclitaxel, erlotinib, methotrexate, and 5-fluorouracil (55–58). Current research extensively demonstrates the substantial involvement of H19 in chemotherapy resistance in malignant tumors. It could potentially serve as a target for treating NPC chemotherapy resistance. For instance, increased H19 expression in paclitaxel-resistant cells, along with H19 silencing combined with paclitaxel, has shown promise in inhibiting NPC progression and enhancing chemotherapy sensitivity (54, 59). Moreover, a sequencing- based construction of LncRNA differential expression profiles associated with NPC paclitaxel resistance was conducted. LncRNAs exhibiting similar expression trends as predicted were further assessed using qRT-PCR. Among NPC cells, n375709 exhibited the highest expression level; its suppression was found to augment the sensitivity of NPC cells to paclitaxel (60). CCAT1, or colon cancer-associated transcript 1, also known as cancer-associated region LncRNA-5 or CCAT1-S, localizes to chromosome 8q24.2 and plays a critical role in biological processes such as invasion and drug resistance (61). In paclitaxel-resistant NPC tissues, CCAT1 is notably overexpressed, indicating its involvement in paclitaxel resistance. Investigations revealed that silencing CCAT1 can inhibit the target gene CPEB2 through sponge miR-181a, thereby enhancing the sensitivity of NPC cells to paclitaxel. This suggests that CCAT1 holds potential as a therapeutic target for NPC (25). At present, there have been many studies on the mechanism of IncRNA in drug resistance of malignant tumors, which is not only related to apoptosis, autophagy, control of carcinogenic signaling pathway, promotion of EMT, but also related to the regulation of cancer stem cells (CSCs) (62). CSCs play an important role in the progression and drug resistance of malignant tumors, and are a small subgroup of tumor cells. It has the ability of self-renewal, increasing DNA repair, body maintenance, drug efflux and redox (63). It has been confirmed that LncRNA is related to CSCs, and chemoradiotherapy can promote the unique self-renewal ability of CSCs through the production of cytokines and DNA repair mechanisms. For example, H19 has been found to participate in the regulation of CSCs, thereby regulating the sensitivity to chemotherapy (64, 65).

## 3 Role of LncRNA in NPC radiotherapy resistance

Considering the specific anatomy of NPC and its sensitivity to ionizing radiation, the primary treatment for NPC is radiotherapy (33). Resistance to radiation stands as a significant contributor to the failure of NPC treatment (66). The core of radiotherapy lies in causing DNA damage, potentially inducing cell apoptosis through radiation exposure (67). However, radiotherapy might trigger mechanisms that repair DNA damage, ultimately bolstering resistance to this form of treatment (68). Recent evidence increasingly supports the pivotal role of LncRNA in NPC radioresistance, suggesting LncRNA as a potential target for enhancing radiotherapy sensitivity and improving the 5-year survival rate of patients with NPC. The mechanism of LncRNA in radiotherapy resistance is similar to that in chemotherapy resistance. For instance, PVT1, encoded by the human PVT1 gene on chromosome 8q24.21, exhibits high expression in NPC tissues. PVT1 is reported to be correlated with radiation resistance and prognosis. Knocking out PVT1 can reduce PIK3CA expression by sequestering miR-515-5p, inhibit the proliferation of nasopharyngeal carcinoma cells, resist radiation, and promote cell apoptosis, so as to improve the radiotherapy sensitivity and prognosis of patients with NPC (26, 27). Additionally, decreased LncRNA levels post-radiotherapy activate cysteinyl aspartate–specific proteinases (caspase), initiating a cascade that triggers caspase 9, caspase 7, and PARP. The binding of caspase 7 and PARP induces apoptosis (69). PVT1, however, inhibits caspase 9, caspase 7, and PARP, impeding apoptosis, thus diminishing NPC sensitivity to radiotherapy and fortifying its resistance (26).Moreover, the downregulation of LINC01140 in NPC cells is closely related to NPC radiation resistance, cancer cell proliferation and apoptosis. It regulates ZNF621 expression by competitively binding miR-452-5p, thereby influencing NPC cell radiosensitivity. Hence, LINC01140 is a potential therapeutic target for NPC (28). Linc00312 exhibits dysregulation and reduced expression in NPC, linked to its role in NPC progression, drug resistance, short-term efficacy, and OS (70). Upregulating Linc00312 enhances NPC sensitivity to radiotherapy by inhibiting radiation-induced signal transduction pathways and DNA damage repair–related protein expression (29, 30). Aberrant expression of PTPRG-AS1, observed in various malignant tumors, including NPC, demonstrates its effect on radiosensitivity. For example, PTPRG-AS1, as a ceRNA of miR-124-3p, regulates the LHX2 target gene, affects the proliferation and apoptosis of NPC cells, and regulates the radiotherapy sensitivity of NPC cells (31). LncRNA can also affect the viability of malignant tumor cells. Furthermore, PTPRG-AS1 binds specifically to miR-194-3p, thereby affecting the target gene PRC1, regulating the activity and apoptosis of NPC cells, and regulating the sensitivity of nasopharyngeal carcinoma cells to radiotherapy (32). CASC19, another noteworthy LncRNA encoded by chromosome 8q24.21, shows high expression in various malignant tumors, including NPC. It participates in proliferation, radioresistance, invasion, and other processes, signifying its potential as a therapeutic target and biomarker for NPC (71). CASC19 expression is closely related to the resistance of NPC cells to radiotherapy. It regulates NPC sensitivity to radiotherapy by modulating not only AMPK-mTOR and blocking autophagy (33) but also FKBP5 expression, acting as a molecular sponge for miR-340-3p, thereby influencing radiation resistance (34). Because of its inherent resistance to radiation, NPC tends to relapse and metastasize distantly, resulting in a poor prognosis for patients (72). Thus, delving deeper into the molecular mechanisms and actively seeking potential targets for NPC treatment remains an urgent need.

## 4 Conclusion

NPC is one of the most prevalent human tumors, particularly among head and neck tumors. Despite its prevalence, the exact causes and mechanisms triggering NPC remain unclear. Radiotherapy combined with chemotherapy is the primary treatment for NPC. However, while this approach exhibits some efficacy against NPC, the 5-year OS rate for patients with NPC remains notably low due to drug resistance. Additionally, the recurrence and metastasis rates remain considerably high (68). To improve the survival rates and tackle drug resistance and recurrence in NPC, there is an urgent need for further exploration into NPC treatment methods. The role of exosomes in NPC has been a focus of ongoing research. Exosomes, which are tiny membrane vesicles ranging from 40 to 100 nm in diameter, are released when various cells fuse with the plasma membrane. Their contents comprise lipids, nucleic acids, and proteins (73, 74). These exosomes are extensively present in bodily fluids such as blood, saliva, cerebrospinal fluid, and urine, showcasing high biological stability, compatibility, and availability. They play crucial roles in signal transduction, immune regulation, and targeted delivery, among other significant functions (75–80). Exosomes derived from gamma-delta-T cells can counteract the radioresistance observed in NPC stem-like cells. Furthermore, when radiotherapy coincides with exosomes derived from gamma-delta-T cells, it exhibits control over NPC (81). Moreover, the upregulated expression of DDX53 in paclitaxel resistant NPC cells shows a correlation with NPC sensitivity to paclitaxel. DDX53 can be transferred through exosomes, thereby regulating the expression of MDR1 and subsequently influencing NPC sensitivity to paclitaxel (82). In summary, this study offers potential insights into NPC treatment and identifies possible therapeutic targets. Exosomes play a pivotal role in NPC by transmitting crucial information to target cells. Additionally, they affect the tumor microenvironment, contributing to NPC resistance against radiotherapy and chemotherapy. This suggests that exosomes might serve as potential biomarkers in NPC.

Immunotherapy is a vital approach for treating malignant tumors, as it fortifies the immune system against them (83). In the context of NPC, immunotherapy includes immune checkpoint inhibitors (ICIs), adoptive cellular immunotherapy (ACT), and tumor vaccines (84). In 2021, China approved the use of programmed death-1/programmed death-ligand 1 (PD-1/PD-L1) blocking ICIs for NPC treatment (85). When the T-cell receptor (TCR) signaling is activated, PD-1 is induced on the T cell, impairing their function. PD-1 then binds to the PD-L1 ligand on cell membranes, facilitating immune evasion and tumor development (83). PD-1 inhibitors block the PD-L1 and PD-1 binding on T-cell surfaces, thereby inhibiting NPC progression. PD-L1 carried by NPC cell–derived exosomes is an immune-related protein that binds to PD-1 on the surface of CD8+T cells, diminishing immune cell activity, fostering NPC immune escape, and advancing NPC occurrence and progression (86). Hyperthermia’s ability to regulate immune responses, bolster radiation resistance, eliminate tumors through heat, impede NPC metastasis and recurrence, and in conjunction with ICIs, elevate 5-year survival rates in patients with NPC (87). ACT employs a patient’s T cells and other structures to combat malignant tumors (88). This encompasses tumor-infiltrating lymphocytes, engineered TCRs, chimeric antigen receptor T cells, and natural killer cell therapies (89, 90). Biologically active immune effector cells are isolated from the patient, multiplied significantly, enhanced in cytotoxic function, or transformed into antiviral cells. Subsequently, these cells are reintroduced into the patient to exert antitumor effects (89). A retrospective analysis involving 38 patients with NPC in a Phase II trial showcased a higher overall response rate when chemotherapy with gemcitabine and carboplatin was combined with the adoptive transfer of six autoamplified Epstein-Barr virus–specific T cells than chemotherapy alone for NPC (91). Moreover, NPC vaccines substantially decreased NPC incidence (92). Glycoproteins on the surface of Epstein-Barr virus cells, such as gp350, gH/gL, and gp42, among others, can be used as vaccine targets. Notably, gp350 is distributed abundantly and holds promise as the most potential vaccine immunogen (93). gp350 has long been acknowledged as an Epstein-Barr virus vaccine candidate for both nonhuman primates and humans (94). A prospective cohort analysis underlines the role of Epstein-Barr virus–neutralizing antibodies, glycoprotein antibodies, and anti-EBNA1 IgA in reducing NPC risk. High levels of anti-GP350 antibodies and B- cell–neutralizing antibodies inhibit infection, suggesting their potential in vaccine development to curtail NPC prevalence (95). Epstein-Barr virus–encoded latent membrane protein 2 (LMP2) antigen can be used as a therapeutic target for NPC. The development of lipid-based LMP2-mRNA (mLMP2) vaccines efficiently expresses LMP2 for NPC immunotherapy (96).

Increasing evidence highlights the dysregulation of LncRNAs in NPC, closely linking them to tumor progression (97). This study summarizes key LncRNAs significant in NPC (**Table 1**). Understanding the mechanisms and biology of LncRNAs in NPC suggests their potential as diagnostic and therapeutic targets. For instance, SNHG14, highly expressed in NPC cells, interacts with miR-5590-3p and elevates the expression of ZEB1, leading to increased programmed cell death receptor-1 (PD-L1) expression and promoting NPC’s epithelial-mesenchymal transition (EMT). Yet, research indicates that nano-coated si-SNHG14 downregulates PD-L1 expression, diminishes EMT, and impedes NPC progression, offering a new potential target for NPC immunotherapy (98). To enhance the efficacy of chemoradiotherapy for NPC, RGD-targeted platinum-based nanoparticles (RGD-PtNPs, termed RPNs) have been developed. RPNs, through RGD, are absorbed by NPC cells, augmenting cisplatin’s effect. Additionally, RGD nanoparticles bind to RGD receptors in cancer cells, effectively targeting NPC (99, 100). Targeted ligands, such as pH and redox dual stimulation-responsive folate-targeted folic acid–β-cyclodextrin–hyperbranched poly (amido amine) (FA-DS-PAAs) nanocarriers, are currently used in precision-targeted therapy for NPC by co-delivering docetaxel and tissue factor pathway inhibitor 2 (TFPI-2) (101). Drug resistance significantly affects the low 5-year survival rate of patients with NPC. Ongoing studies elucidating the intricate relationship between LncRNAs and drug resistance underscore the potential of LncRNAs as treatment targets for NPC. Further clinical investigations are warranted to enhance the 5-year survival rate of patients with NPC.

## Author contributions

YY: Writing – original draft. QY: Writing – review & editing. WT: Writing – review & editing. YM: Writing – review & editing. JD: Writing – review & editing. GY: Conceptualization, Writing – review & editing. YF: Conceptualization, Writing – review & editing.
